# Epidemiology and Management of Chronic Thromboembolic Pulmonary Hypertension in Greece. Real-World Data from the Hellenic Pulmonary Hypertension Registry (HOPE)

**DOI:** 10.3390/jcm10194547

**Published:** 2021-09-30

**Authors:** Eftychia Demerouti, Panagiotis Karyofyllis, Vassilios Voudris, Maria Boutsikou, George Anastasiadis, Anastasia Anthi, Alexandra Arvanitaki, George Athanassopoulos, Aikaterini Avgeropoulou, Styliani Brili, Christos Feloukidis, Frantzeska Frantzeskaki, George Karatasakis, Haralambos Karvounis, Dimitrios Konstantonis, Ioanna Mitrouska, Sophia Mouratoglou, Katerina K. Naka, Stylianos E. Orfanos, Evangelia Panagiotidou, Georgia Pitsiou, Antonios Pitsis, Vagia Stamatopoulou, Ioannis Stanopoulos, Adina Thomaidis, Iraklis Tsangaris, Dimitrios Tsiapras, George Giannakoulas, Athanassios Manginas

**Affiliations:** 1 Cardiology Department, Onassis Cardiac Surgery Center, 17674 Athens, Greece; pakar768@yahoo.gr (P.K.); vvoudris@otenet.gr (V.V.); george.d.athanassopoulos@gmail.com (G.A.); georgekar2001@yahoo.com (G.K.); dtsiapras@hotmail.com (D.T.); 2 Cardiology Department, Mediterraneo Hospital, 16675 Athens, Greece; boutsikoum@gmail.com (M.B.); nassoseft@yahoo.com (A.M.); 3 Cardiology Department, Laikon Hospital, 11527 Athens, Greece; paokanas@gmail.com; 4 Multidisciplinary Pulmonary Hypertension Center, Attikon University General Hospital, 12462 Athens, Greece; anastasia.anthi1@gmail.com (A.A.); ffrantzeska@gmail.com (F.F.); dkonstantonis@gmail.com (D.K.); stylianosorfanosuoa@gmail.com (S.E.O.); itsagkaris@med.uoa.gr (I.T.); 5 Cardiology Department, AHEPA University Hospital, Aristotle University of Thessaloniki, 54621 Thessaloniki, Greece; alexandra.arvanit@gmail.com (A.A.); cfelouk@gmail.com (C.F.); hkarvounis@auth.gr (H.K.); s_mouratoglou@yahoo.gr (S.M.); g.giannakoulas@gmail.com (G.G.); 6 Cardiology Department, Hippokration General Hospital, 11527 Athens, Greece; catherine.avgeropoulou@gmail.com (A.A.); stlbrili@gmail.com (S.B.); 7 Department of Intensive Care Medicine, University Hospital of Heraklion, 71500 Heraklion, Greece; mitrouska@med.uoc.gr (I.M.); vagiastamatopoulou@gmail.com (V.S.); 8 2nd Department of Cardiology, University Hospital of Ioannina, 45500 Ioannina, Greece; drkknaka@gmail.com; 9 1st Department of Critical Care, Pulmonary Hypertension Clinic, Enangelismos General Hospital, 10676 Athens, Greece; 10 Respiratory Failure Unit, “G. Papanikolaou” Hospital, 57010 Thessaloniki, Greece; evangeliapanagiotidou@gmail.com (E.P.); gpitsiou@yahoo.gr (G.P.); istan@otenet.gr (I.S.); 11 Interbalkan Medical Center, 55535 Thessaloniki, Greece; info@apitsis.gr; 12 Cardiology Department, Democritus University of Thrace, 69100 Alexandroupolis, Greece; adinathomaidis@gmail.com

**Keywords:** pulmonary hypertension, chronic thromboembolic pulmonary hypertension, balloon pulmonary angioplasty, pulmonary endarterectomy, registry

## Abstract

Chronic Thromboembolic Pulmonary Hypertension (CTEPH) is a rare disease with poor prognosis if left untreated, characterized by pulmonary vascular bed obstruction due to unresolving thromboembolic material. The Hellenic pulmonary hypertension registry (HOPE) was launched in Greece in early 2015 and enrolls patients from all pulmonary hypertension subgroups in Greece. In total, 98 patients with CTEPH were enrolled from January 2015 until November 2019. Of these patients, 55.1% represented incident population, 50% were classified in the World Health Organization functional class II and 49% had a history of acute pulmonary embolism. The median values of pulmonary vascular resistance (PVR) and cardiac index were 7.4 (4.8) WU and 2.4 (1.0) L/min/m^2^, respectively, the mean diffusing capacity for carbon monoxide was 74.8 ± 20.6%, the median 6-minute walk distance was 347 (220) meters and the median value of N Terminal-pro brain natriuretic peptide was 506.0 (1450.0) pg/mL. In total, 60.2% of the patients were under pulmonary arterial hypertension-targeted therapy at the time of enrolment; specifically, riociguat was received by 35.7% of the patients and combination therapy was the preferred strategy for 16% of the patients. In total, 74 patients were evaluated for pulmonary endarterectomy (PEA), 34 (45.9%) were assessed as operable but only 23 of those (31.1%) finally underwent PEA. The remaining 40 patients were ineligible for PEA according to the operability assessment and 13 (17.6%) of them underwent balloon pulmonary angioplasty. The age of the non-operable patients was significantly higher than the operable patients (*p* < 0.001), while there was no significant difference with regard to the history of coagulopathies between the operable and non-operable patients (*p* = 0.33).

## 1. Introduction

Chronic thromboembolic pulmonary hypertension (CTEPH) is defined by increased pulmonary vascular resistance (PVR) due to unresolved thromboembolic material in the pulmonary vascular bed, leading to right ventricular failure and death [1]. According to the pulmonary hypertension (PH) Guidelines [2], CTEPH is a precapillary PH according to the hemodynamic definition, as the mean pulmonary arterial pressure (mPAP) is more than 25 mmHg and the pulmonary arterial wedge pressure (PAWP) lower than 15 mmHg. A Task Force from the sixth World Symposium on PH recently proposed [3] a new definition of pre-capillary PH, with mPAP > 20 mmHg, PAWP < 15 mmHg and pulmonary vascular resistance (PVR) > 3 Wood Units. The present observational cohort study provides information on CTEPH patients’ baseline data (at the time of first visit of patients at the PH centers) and on their therapeutic approach at baseline. The patients are enrolled in the Hellenic Pulmonary Hypertension Registry (HOPE).

## 2. Methods

The Hellenic Pulmonary Hypertension Registry (HOPE) is a PH registry launched in January 2015 and continues to enroll patients, mainly with Pulmonary Arterial Hypertension PAH [4] and CTEPH. The HOPE registry has been approved by the Institutional Review Board of each one of the ten participating PH expert centers in Greece according to the Declaration of Helsinki. All patients provided written informed consent for their inclusion in the study. Documentation has been Internet-based (PAH tool by Inovultus Lda, Portugal) and includes demographics, type of PH according to the European Guidelines, comorbidities, clinical symptoms and signs, World Heart Organization (WHO) functional class (FC), 6-minute walk distance (6-MWD), N-terminal pro-brain natriuretic peptide (NT-proBNP) serum levels, hemodynamic parameters, computed tomography data, ventilation/perfusion scintigraphy (V/Q Lung scanning) and detailed information about medications for PH, including supportive measures, such as oxygen therapy and anticoagulation, as well as PAH-specific medical therapy. Pulmonary endarterectomy (PEA) or balloon pulmonary angioplasty (BPA) were also included as therapeutic strategies. The diagnosis of CTEPH was established after excluding other causes of PH according to the current guidelines [2]. CTEPH was defined by the following: (1) mPAP > 25 mmHg with PAWP ≤ 15 mmHg as measured by Right Heart Catheterization (RHC), (2) abnormal ventilation–perfusion (VQ) scan, pulmonary angiogram, computed tomography pulmonary angiography (CTPA) or magnetic resonance pulmonary angiography (MRPA) confirming chronic thromboembolic obstructions as described in European Guidelines [2] and (3) after at least three months of adequate anticoagulation therapy.

The participating centers enter all their eligible patients on a consecutive basis. Data are collected at the time of first visit of patients at the PH centers and at least in 6-month intervals or whenever the patient has a predefined clinical event such as death, transplantation, PAH-related hospitalization, deterioration in FC, any unscheduled change in PAH therapy, or other serious adverse events.

The cut-off date for the data analysis of the present study was 18 November 2019. Inclusion criteria for this study were a diagnosis of CTEPH according to the definitions of the 2015 guidelines [5], age > 14 years and availability of data from RHC.

### Statistical Methods

Data were presented as mean ± standard deviation for continuous variables with normal distribution, and as median and interquartile range for non-normally distributed variables. Categorical variables were presented as frequencies and percentages (%). Continuous variables were compared using the *t*-test for independent samples or the Mann-Whitney U test, while the chi-square test or the Fisher exact test was used to assess categorical variables. For multiple comparisons, one-way Analysis of Variance ANOVA or the Kruskal-Wallis test with post hoc analysis was used as appropriate. A *p*-value < 0.05 was considered statistically significant in this study. Data were analyzed using the SPSS version 23.0. (IBM SPSS Statistics for Windows, Version 23.0. Armonk, NY, USA).

## 3. Results

### Baseline Characteristics

Between January 2015 and November 2019, 98 patients with CTEPH were enrolled from 10 Greek PH centers. Of note, three expert centers evaluate and manage the majority of CTEPH patients (79.6%). Table 1 shows the baseline demographic characteristics and the comorbidities. At the time of the patients’ enrolment, 54 (55.1%) were incident patients, 58 (59.2%) were women, while the median age was 60 ± 16 years. In terms of comorbidities, arterial hypertension was the most prevalent (32.7%) underlying condition. The main presenting symptoms and functional assessment are presented in Table 2. Half of the patients were classified in functional class II according to the WHO classification, with dyspnea being the most frequent symptom (85.7%).

Of the patients, 48 (49%) had a history of acute pulmonary embolism (PE) and 30 (30.6%) deep vein thrombosis (DVT). A minority had a history of coagulopathy (*n* = 11, 11.2%) and 13 (13.3%) patients had a history of splenectomy.

Hemodynamic parameters were reported and the median value of mPAP, PVR and C.I. was 44.5 (15.3) mmHg, 7.4 WU (4.8) and 2.4 (1.0) L/min/m^2^, respectively (Table 3). The mean diffusing capacity for carbon monoxide (DLCO) was 74.8 ± 20.6%, measured in 39 (39.8%) patients. With regard to imaging, echocardiographic parameters are reported in Table 3. The mean value of peak tricuspid regurgitation velocity was 3.9 ± 0.7 m/s. V/Q lung scanning was performed in 60 (61.2%) patients and the rest of the patients underwent CTPA (38 patients). A total of eight patients from the entire population underwent V/Q lung scanning and CTPA and 40 patients underwent invasive Pulmonary Angiography. On CTPA, 18 patients (18.4%) had central thrombi, 11 (52.4%) peripheral and 9 (9.2%) mural thrombi.

## 4. Therapeutic Strategies

Anticoagulants were used in all the patients, predominantly vitamin-K antagonists (63.3%), while the remaining received non-vitamin K oral anticoagulants (NOACs) (Table 4). Of the patients, 60.2% were under specific PAH-targeted therapy at the time of their enrolment in the registry, and 43.9% were on monotherapy. Riociguat was received by 35.7% of the patients, prostanoid analogues and IP receptor antagonist in 11 (7.2%) patients and combination PAH-targeted drug therapy was the preferred strategy for 16% of the patients.

**Figure 1:**
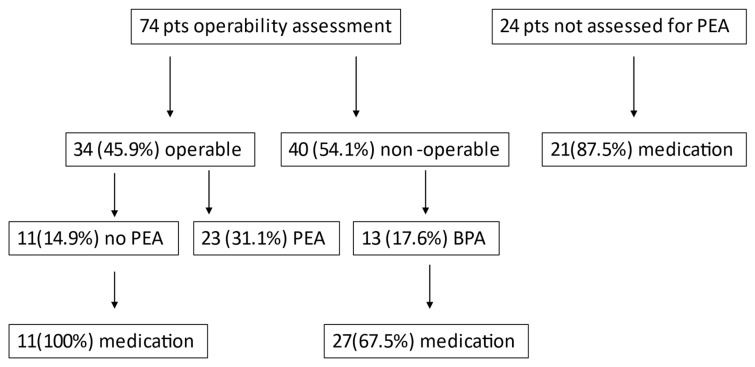
Therapeutic Management. Pts: patients, PEA: Pulmonary Endarterectomy, BPA: Balloon Pulmonary Angioplasty.

Until the database closure for analysis, seventy-four patients were evaluated for PEA, 34 (45.9%) were assessed as operable and finally 23 (31.1%) underwent PEA. Forty patients (54.1%) were ineligible for PEA according to their operability assessment and 13 of these (17.6%) underwent BPA (Figure 1). Twenty-one of the patients (87.5%) who had not been evaluated for PEA, 27 non-operable patients who did not undergo BPA and 11 patients who refused PEA were under specific-PAH drug therapy.

## 5. Discussion

### Comparison between Operable and Non-Operable Patients

The age of the non-operable patients was significantly higher than the operable patients and they were clinically and functionally more compromised based on the WHO classification and 6-MWD (Table 5). There were no significant differences between the hemodynamic parameters. There were also no differences with regard to the history of coagulopathies (antiphospholipid syndrome, thrombotic thrombocytopenic purpura, thrombocytosis) between the operable and non-operable patients (*p* = 0.33). As expected, the operable patients had a more frequent central thrombi in CTPA (17.6%) compared to the non-operable patients (*p* = 0.008).

## 6. Discussion

This is the first National Registry on CTEPH in Greece, supported by 10 expert PH centers nationally, providing baseline information on demographics, epidemiology, clinical characteristics and therapeutic management of the Greek patient population with CTEPH. This retrospective study combines the prevalent and incident population. Three previous registries reported the prevalence of CTEPH ranging from 3.2 cases/MI in Spain [6] to 9 cases/MI in Sweden [7] and 15.7 cases/MI in the Latvian registry [8]. According to the 2019 Eurostat Data [9], the population in Greece was 10,724,599. The HOPE registry seems to have captured 72.5% of CTEPH Greek patients, based on a prevalence of 12.63 cases/MI (mean value of prevalence in the three registries).

The majority of the patients in the HOPE registry are women and a history of PE/VTE is obvious in half of the patients, in concordance with the recently published data on the International CTEPH Registry [10], where the median age was 62 years for women and 63 for men and an episode of VTE was present in more than 70% of the enrolled patients. The Japanese registry [11] revealed 50.4% and 37.2% of patients had a history of deep vein thrombosis and acute pulmonary embolism, respectively. The corresponding percentages were 56.1% and 74.8% in the large European database [12] and in concordance with this registry, a large proportion of our patients had a history of PE (49%)) and DVT (30.6%) and a significant number had a history of splenectomy, perhaps due to the high prevalence of haemoglobinopathies in Greece. There were also no differences with regard to the history of coagulopathies between the operable and non-operable patients (*p* = 0.33), similarly to the International CTEPH registry [12].

In comparison with the published CTEPH registries [9,12,13,14,15,16,17], the patients in the HOPE registry were not significantly impaired clinically and hemodynamically, perhaps due to the inclusion of both incident and prevalent cases. The majority of the patients presented in the WHO FC II and III at the time of their enrolment, and the median CI was 2.4 L/min/m^2^. In the European Registries, the predominant WHO functional class was class III and the median CI was lower than 2.2 L/min/m^2^. In the International CTEPH Registry [12] and HOPE registry, the median CIs (L/min/m^2^) were 2.2 and 2.4 and the median PVRs (WU) were 8.9 and 7.4, respectively. The median value of PVR for the operable patients in our cohort is 9.1 WU in concordance with the International Registry in which the median PVR was 9 WU for operable patients. Of note, in the International CTEPH registry, 43% of non-operable patients had proximal lesions, underlining that there is not always concordance between hemodynamic parameters and operability.

It is notable that multimodality imaging is not provided in all centers in Greece. Invasive PA is performed in three centers in Greece and Lung V/Q scanning only in one of them. We also found distinct regional differences in management decisions concerning the treatment modalities of PEA. It is also of paramount importance that not all the ineligible patients for PEA are evaluated for BPA in expert BPA centers in our country.

CTEPH represents the only curable form of PH [12]. PEA is the treatment of choice, however not all patients are eligible for surgical treatment. In patients who are deemed non-operable or in the case of recurrent PH after PEA, specific PAH-drug therapy is indicated with or without BPA according to an algorithm proposed by the sixth WSPH Task Force on PH diagnosis and treatment [18]. In the large European Registry, 62.9% of patients were considered operable and 56.8% finally underwent PEA. In a recently published worldwide prospective CTEPH registry [19], 1010 patients were enrolled and 64.3% were considered for PEA, 19.1% for BPA and 2% for both PEA and BPA. In Europe and America and other countries, 72% were deemed suitable for PEA, whereas in Japan, 70% of patients were offered BPA as the first choice, highlighting a noticeable difference in therapeutic approaches in Japan compared with Europe. In our cohort, among the patients assessed for PEA, 45.9% were judged operable, 31.1% underwent PEA and the rest remained on drug therapy or underwent BPA. The decision on PEA, based on technical operability, comorbidities and patient preference, was made by the expert PH centers. A total of 11 patients who were deemed operable, refused to be operated on. The main reason for the low surgical rate may be explained by the lack of an officially designated PEA center in Greece; therefore, some of the operable CTEPH patients undergo surgery abroad [20]. A total of 40 patients (54% of those who were assessed for PEA) were considered inoperable, 12 (30%) due to comorbidities and 28 (70%) patients due to distal involvement.

At present, we point to the importance of active BPA programs in Greece, as 17.6% of non-operable patients and 5.4% of operable patients did undergo BPA, a promising efficacious method, complementary to PEA in the treatment algorithm of CTEPH [21]. BPA is not suitable for all non-operable patients. The optimal selection of patients and lesions is of great importance for a good prognosis, as many complications exist. The therapeutic strategies, efficacy and safety of BPA vary greatly among institutions, and interventionist experience and technical skills. In the recently published worldwide CTEPH registry [18], in the whole population (1010 pts), PEA was performed in 58.7% of patients and BPA in only 17.3%, a proportion similar to our cohort (17.6%).

Pulmonary microvascular disease in CTEPH has provided the rationale for the use of drugs approved for PAH and riociguat has been proven to reduce PH severity and improve functional status for non-operable patients or those with recurrent PH after PEA [22]. In the International CTEPH Registry [12], 37.9% of patients received PAH specific drugs and 4% combination therapy. In our study, 60.2% of patients were under drug treatment. This difference may exist due to the mixed population (prevalent and incident patients) in the HOPE registry; the fact that prevalent patients were already under targeted treatment, which also justifies the high proportion of patients under combination therapy (56.1%); and that data in HOPE were almost 10 years old and retrospective in nature. Although riociguat is the only approved drug for non-operable CTEPH and for persistent PH after PEA, many of our patients received PAH-specific drug therapy other that riociguat at their first assessment, because riociguat became available during early 2015 in Greece. Of note, 74% of those patients were non-operable. However, data are lacking for the patients with medical contraindications or those refusing surgery. Using medical therapy as a “bridge to PEA” is more controversial, and is felt to delay timely surgical referral and, therefore, definitive treatment [19]. However, specific PAH-drug therapy may be necessary to be initiated in case of clinical and hemodynamic deterioration until surgery is performed, predominantly in countries where official PEA programs do not exist [5].

In terms of supportive therapy, oral anticoagulants were used in all the patients, since, according to guidelines [2], life-long anticoagulation is recommended in CTEPH patients. NOACs were administered in 36.7% of our patients. A German [23] study recently reported that NOACs are increasingly used in patients with CTEPH, despite the lack of evidence on their use in this specific population. Data suggestive of the appropriate form of anticoagulation therapy in CTEPH are lacking. Traditional anticoagulation with oral vitamin K antagonists is currently recommended and whether the new oral anticoagulants are adequate in CTEPH is currently unknown [18,24].

## 7. Limitations of the Study

The main weakness of this work is the lack of information about the results (PEA results, BPA results, global cohort survival, medically managed patient survival, etc.). However, analyzing the way we are dealing with a clinical entity can help us to improve its management. The lack of electrocardiographic data and data concerning echocardiography for all the included patients and the fact that all data represent the characteristics in the initial assessment represent limitations of the study.

The relatively small sample size can impair the statistical analysis, particularly when comparing various small subgroups. We were unable to capture patients who were never referred to one of the participating centers in the HOPE registry. However, we have to take into consideration that according to the estimated CTEPH prevalence in our country, the HOPE registry seems to capture 72.5% of Greek patients.

Our study refers to a mixture of prevalent and incident patients since the main HOPE registry includes both types of patients. This mixed population could lead to a survival bias in our future analysis.

The initial therapeutic management was studied and data regarding the clinical course and survival over time are still pending. It is a limitation that not all the patients in our cohort were referred for PEA, because many consultant cardiologists based their clinical decision on imaging, clinical situation, comorbidity conditions and hemodynamic characteristics. Importantly, a patient should not be considered inoperable until reviewed by a specialist CTEPH team, including an experienced PEA surgeon. The absence of this team in Greece probably contributes to a suboptimal therapeutic approach. One of the main scopes of this work was to reveal patient management issues that could be corrected through a national CTEPH center.

BPA and PEA were studied as there were data for the patients’ follow-up regarding their invasive therapeutic approach. The registry will continue to assess the clinical evolution of these patients to provide more robust data in the future.

## 8. Conclusions

In conclusion, CTEPH is an increasingly recognized condition worldwide with a potentially curable surgical treatment [2]. Unfortunately, in Greece, not all patients were referred for PEA; furthermore, not all operable patients underwent PEA, reinforcing the need for dedicated PEA centers in Greece, and not all non-operable patients were evaluated for possible BPA in Greek expert centers. The therapeutic process of CTEPH but also the diagnostic management need to be improved globally. All this improvement can be based on the cornerstone recommendation for pulmonary hypertension referral centers [3] mentioning that a referral center should follow at least 50 patients with Pulmonary Arterial Hypertension (PAH) and CTEPH and should receive at least two new referrals per month. The HOPE registry gives us the opportunity, by analyzing, assuming and recognizing our weaknesses, to make more progress toward better management of the disease.

## Figures and Tables

**Table 1:** Baseline demographic and clinical characteristics.

Subjects	*n* = 98
Incident	54 (55.1)
Female	58 (59.2)
Age, years	60 ± 16
BMI, (kg/m^2^)	27.1 (7.0)
Comorbidities	
Arterial hypertension	32 (32.7)
Diabetes	7 (7.1)
Atrial fibrillation	2 (2.0)
CAD	4 (4.1)
Dyslipidemia	9 (9.2)
COPD	11 (11.2)
Hypothyroidism	9 (9.2)
Obesity CKD	23 (23.5) 2 (2.0)
Hematological disease	20 (20.4)
Smoking	27 (27.6)

Categorical variables are presented as frequency and percentage, *n* (%). Continuous variables are presented as mean value ± standard deviation or median value with interquartile range. CTEPH: Chronic Thromboembolic Pulmonary Hypertension; BMI: Body Mass Index; CAD: Coronary Artery Disease; COPD: Chronic Obstructive Pulmonary Disease; CKD: Chronic Kidney Disease.

**Table 2:** Symptoms and Functional Assessment.

*n* (%)	
**Patients**	**98 (100)**
**Symptoms**	
Dyspnea	84 (85.7)
Fatigue	55 (56.1)
Palpitations	20 (20.4)
Chest pain	10 (10.2)
Syncope	3 (3.1)
Hemoptysis	3 (3.1)
RHF	18 (18.4)
**WHO FC**	**98 (100)**
I	7 (7.1)
II	49 (50.0)
III	41 (41.8)
IV	1 (1.0)
**6-MWD, m**	**67 (68.4%)**
347 (220)	

Categorical variables are presented as frequency and percentage, *n* (%). Continuous variables are presented as mean value ± standard deviation or median value with interquartile range. CTEPH: Chronic Thromboembolic Pulmonary Hypertension; RHF: Right Heart Failure; WHO: World Health Organization; FC: Functional Class; 6-MWD: Six-minute Walk Distance.

**Table 3:** Baseline Hemodynamic parameters and Echocardiographic findings.

Subjects	*n* (%)
RHC	98 (100)
mRAP, mmHg	8.0 (5.0)
mPAP, mmHg	44.5 (15.3)
PAWP, mmHg	10.0 (4.5)
CO, L/min	4.3 (1.5)
CI, L/min/m^2^	2.4 (1.0)
PVR, WU	7.4 (4.8)
HR, bpm	78.0 (14.3)
SVO2, %	67.0 (14.0)
Echocardiography	61 (62.2)
RV hypertrophy	23 (23.5)
RVEDD, mm	33.2 ± 12.5
TAPSE, mm	18.5 ± 4.7
TR Vmax, m/s	3.9 ± 0.7
RVSP, mmHg	74.0 ± 22.5

Categorical variables are presented as frequency and percentage, *n* (%). Continuous variables are presented as mean value ± standard deviation or median value with interquartile range. *n*: the absolute count of variables measured in the overall population; RHC: Right heart catheterization; mRAP: mean Right Atrial Pressure; mPAP: mean Pulmonary Artery Pressure; PAWP: Pulmonary Artery Wedge Pressure; CO: Cardiac Output; CI: Cardiac Index; PVR: Pulmonary Vascular Resistance; WU: Wood Units; HR: Heart Rate; bpm: beats per minute; SvO2; oxygen saturation in pulmonary artery; RV: Right Ventricle; RVEDD: Right Ventricular End-Diastolic Diameter; TAPSE: Tricuspid Annular Plane Systolic Excursion; TR Vmax: maximal velocity of tricuspid regurgitation; RVSP: Right Ventricular Systolic Pressure.

**Table 4:** Baseline Supportive and Targeted-PAH therapy.

	*n* (%)	CTEPH
Subjects	*n* = 98	Baseline
Diuretics		49 (50.0)
OAC		98 (100)
NOAC		36 (36.7)
Warfarin/acenocumarol		62 (63.3)
Targeted PAH Therapy		
No therapy		39 (39.8)
Monotherapy		43(43.9)
Double Combination		14 (14.3)
Triple Combination		2 (2.0)
Ca channel antagonists		5(5.1)
PDE5i		10 (10.2)
sGC		35 (35.7)
ERA		22 (22.4)
Prostacyclin analogues		10(10.1)
IP antagonists (selexipag)		1 (1.0)
iv epoprostenol		2 (2.0)
sc treprostinil		2 (2.0)
inh iloprost		6 (6.1)
Double Combination		
PDE5i + ERA		3 (3.1)
PDE5i + Prostanoid		1 (1.0)
ERA + Prostanoid		5 (5.1)
Riociguat + PDE5i		1 (1.0)
Riociguat + ERA		4 (4.1)

Categorical variables are presented as frequency and percentage, *n* (%). Continuous variables are presented as mean value ± standard deviation or median value with interquartile range. *n*: the absolute count of variables measured in the overall population; CTEPH: Chronic Thromboembolic Pulmonary Hypertension; OAC: Oral Anticoagulant; NOAC: New Oral Anticoagulant; CCBs: Calcium Channel Blockers; PDE5i: Phosphodiesterase type 5 inhibitors; sGC: Guanylate Cyclase stimulator; ERA: Endothelin Receptor Agonists.

**Table 5:** Clinical, hemodynamic data and therapeutic management of patients with CTEPH according to their operability. Categorical variables are presented as frequency and percentage, *n* (%).

*n* = 74	Operable *n* = 34	Non-Operable *n* = 40	*p*-Value *
Subjects	34 (45.9)	40 (54.1)	
Female	16 (21.6)	27 (36.5)	0.099
Age, years	53 ± 13	63 ± 15	<0.0001
BMI, (kg/m^2^)	27.0 (8.2)	27.1 (5.7)	0.542
WHO FC			
I	5 (14.7)	1 (2.5)	0.037
II	18 (52.9)	16 (40)	
III	10 (29.4)	23 (57.5)	
IV	1 (2.9)	0	
6-MWD, m	420 (202)	347 (206)	0.278
NT-proBNP, pg/mL	570 (1706)	814.0 (1949)	1.0
Right heart catheterization			
mRAP, mmHg	8.0 (6.8)	8.5 (8.0)	0.557
mPAP, mmHg	47.5 (20.5)	44 (22)	0.792
PAWP, mmHg	10.0 (4.8)	12 (3.0)	0.758
CO, L/min	4.2 ± 0.8	4.8 ± 1.7	0.385
CI, L/min/m^2^	2.3 ± 0.4	2.6 ± 0.9	0.357
PVR, WU	9.1 (5.7)	6.8 (6.7)	0.536
HR, bpm	74 ± 8	78 ± 10	0.091
SVO2, %	64.9 ± 8.9	63.6 ± 10	0.375
V/Q scintigraphy (abnormal)	14 (18.9)	13 (17.6)	0.511
Chest CTPA	19 (55.9)	13 (32.5)	0.062
Central thrombi	13 (38.2)	2 (5)	0.008
Peripheral thrombi	10 (29.4)	10 (25)	0.686
Pulmonary Angiography	19 (55.9)	21 (52.5)	0.818
Supportive Therapy			
Oxygen therapy	10 (29.4)	15 (37.5)	0.622
Diuretics	14 (18.9)	21 (52.5)	0.359
OAC	27 (79.4)	23 (57.5)	0.051

Continuous variables are presented as mean value ± standard deviation (SD) or median value with interquartile range (IQR). * Statistical significance between operable and inoperable patients with CTEPH: *p* < 0.05. CTEPH: Chronic Thromboembolic Pulmonary Hypertension; BMI: Body Mass Index; WHO: World Health Organization; FC: Functional Class; 6-MWD: Six-minute Walk Distance; NT-proBNP: N-terminal pro-brain natriuretic peptide; mRAP: mean Right Atrial Pressure; mPAP: mean Pulmonary Artery Pressure; PAWP: Pulmonary Artery Wedge Pressure; CO: Cardiac Output; CI: Cardiac Index; PVR: Pulmonary Vascular Resistance; WU: Wood Units; HR: Heart Rate; bpm: beats per minute; SvO2: oxygen saturation in pulmonary artery; CTPA: Combuted Tomography Pulmonary Angiography, OAC: Oral Anticoagulant.
